# Left circumflex artery occlusion during aortic valvuloplasty in a young patient with bicuspid aortic valve: a case report

**DOI:** 10.1186/s40981-025-00806-8

**Published:** 2025-07-24

**Authors:** Yutaro Otsuka, Tsunehisa Tsubokawa

**Affiliations:** Department of Anesthesiology, The Jikei University School of Medicine, Tokyo, Japan

**Keywords:** Aortic valvuloplasty, Bicuspid aortic valve, Coronary artery injury, Left circumflex artery, Transesophageal echocardiography

## Abstract

**Background:**

Aortic valvuloplasty serves as a valve-preserving alternative to aortic valve replacement and offers advantages in younger patients. However, intraoperative complications associated with this technique have rarely been reported.

**Case presentation:**

A 15-year-old male with severe aortic regurgitation due to a congenital bicuspid aortic valve underwent aortic valvuloplasty. During separation from cardiopulmonary bypass, ST-segment elevation was noted on electrocardiography, and transesophageal echocardiography (TEE) revealed regional wall motion abnormalities. Color Doppler imaging revealed absent flow in the left circumflex artery (LCx). The anesthesiology team promptly alerted the surgeons, and LCx occlusion due to suture annuloplasty was suspected. Removal of the implicated suture restored flow and stabilized hemodynamics.

**Conclusions:**

Systematic intraoperative assessment and documentation of coronary flow via TEE are instrumental in diagnosing coronary artery occlusion. Moreover, mutual trust and clear, timely communication between anesthesiologists and surgeons are essential to maintaining surgical safety.

**Supplementary Information:**

The online version contains supplementary material available at 10.1186/s40981-025-00806-8.

## Background

Surgical treatment of aortic regurgitation in younger patients typically entails either aortic valve replacement or valve-preserving repair via aortic valvuloplasty. The former necessitates lifelong anticoagulation and carries well-documented risks, including bleeding, thromboembolic events, and prosthetic valve endocarditis [1, 2]. In contrast, aortic valvuloplasty preserves the native valve architecture and may substantially mitigate the long-term risks associated with prosthetic valve implantation [1–4]. However, its broader adoption remains constrained by the absence of standardized surgical protocols, inherent technical complexity, and unresolved concerns regarding the long-term durability of the reconstructed valve, which may ultimately necessitate reoperation [5, 6]. As a result, intraoperative complications related to valvuloplasty have been rarely documented in the literature.

Herein, we report a case in which transesophageal echocardiography (TEE) detected left circumflex artery (LCx) occlusion during aortic valvuloplasty, and echocardiographic findings guided prompt surgical resolution of the obstruction.

Written informed consent for publication of this case report and all accompanying images and videos was obtained from the patient’s legal guardians.

## Case presentation

A 15-year-old male (height 164.0 cm; weight 50.0 kg) with severe aortic regurgitation due to a congenital bicuspid aortic valve was referred to our institution for definitive surgical management. He had no notable past medical history. Preoperative transthoracic echocardiography revealed severe aortic regurgitation, accompanied by mild aortic stenosis, mild mitral regurgitation, and left ventricular enlargement. The aortic valve was identified as a bicuspid valve with right–noncoronary cusp fusion (Sievers type I), exhibiting pronounced commissural asymmetry (commissural angle 144°). No regional wall motion abnormalities (RWMA) were observed, and the left ventricular ejection fraction was preserved at 67.5%, with no evidence of diastolic dysfunction. Electrocardiography demonstrated normal sinus rhythm at a rate of 59 beats per minute. Coronary computed tomography angiography showed no evidence of coronary artery stenosis. Although mild hypoplasia of the right coronary artery was present, no anatomical anomalies were identified in the left coronary artery.

General anesthesia was induced using propofol, remifentanil, and rocuronium, and maintained using total intravenous anesthesia with propofol. Intraoperative TEE confirmed the preoperative findings and demonstrated preserved coronary flow in the left main trunk, proximal left anterior descending artery (LAD), proximal LCx, right coronary ostium, right posterior descending artery, and right atrioventricular branch.

A median sternotomy was performed, and cardiopulmonary bypass (CPB) was initiated via right femoral arterial cannulation and right atrial venous drainage. Myocardial arrest was induced using a combination of retrograde and selective antegrade cardioplegia, administered at 20-min intervals. In view of the favorable valvular anatomy, aortic valvuloplasty was undertaken. Suture annuloplasty was initially performed, followed by central plication of the fused cusp. When ventricular-side bulging of the cusp was identified, direct repair was carried out, and sinus plication was subsequently incorporated to optimize valve configuration. The procedure also included replacement of the ascending aorta, thereby completing the anatomical reconstruction of the aortic valve complex.

**Fig. 1:**
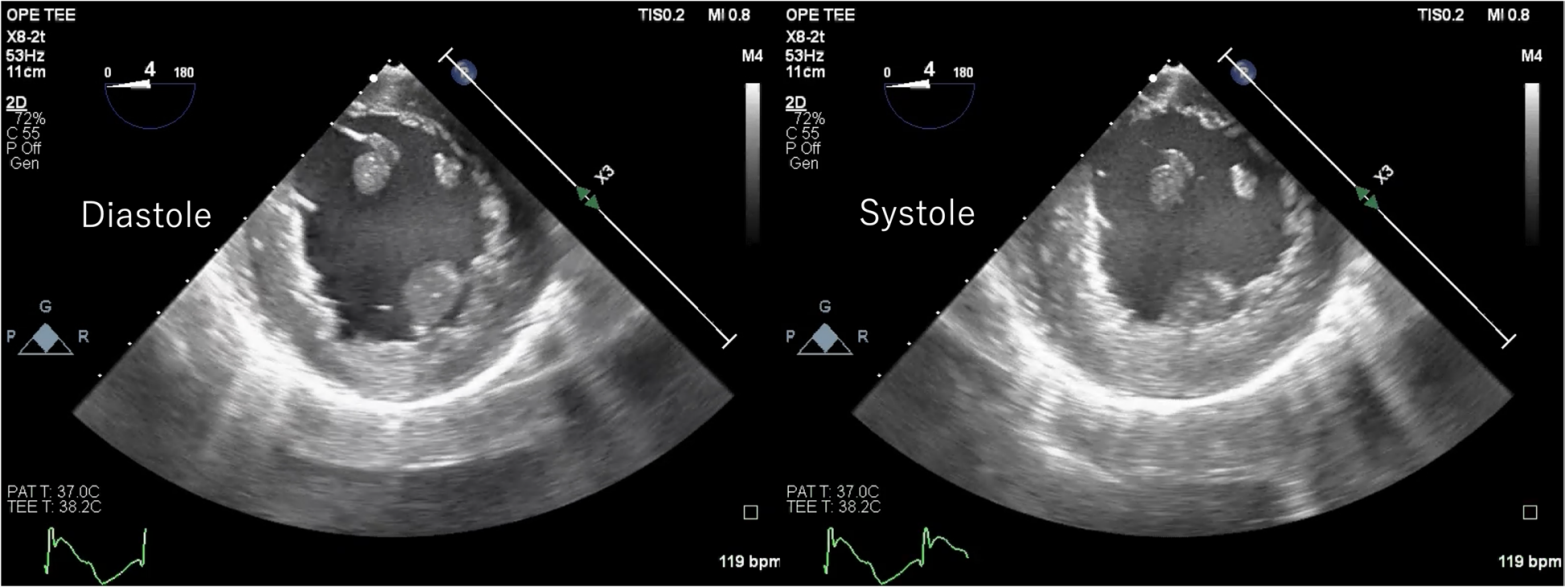
Regional wall motion abnormality observed on a mid-esophageal short-axis view. Transesophageal echocardiographic images obtained during diastole (left) and systole (right) show a regional wall motion abnormality extending from the lateral to the inferior wall

**Fig. 2:**
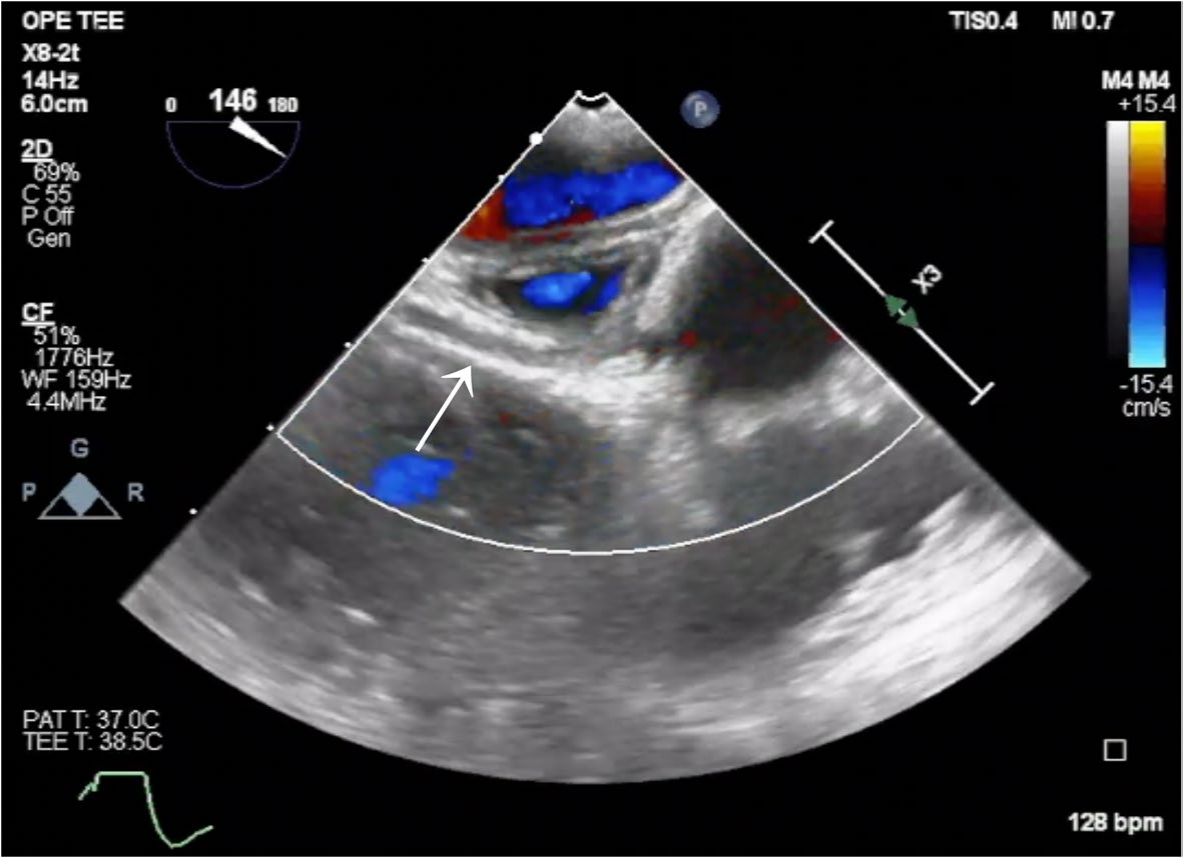
Absence of flow in the left circumflex artery Color Doppler image obtained using transesophageal echocardiography. The solid arrow indicates the left circumflex artery (LCx), where no color flow is observed

**Fig. 3:**
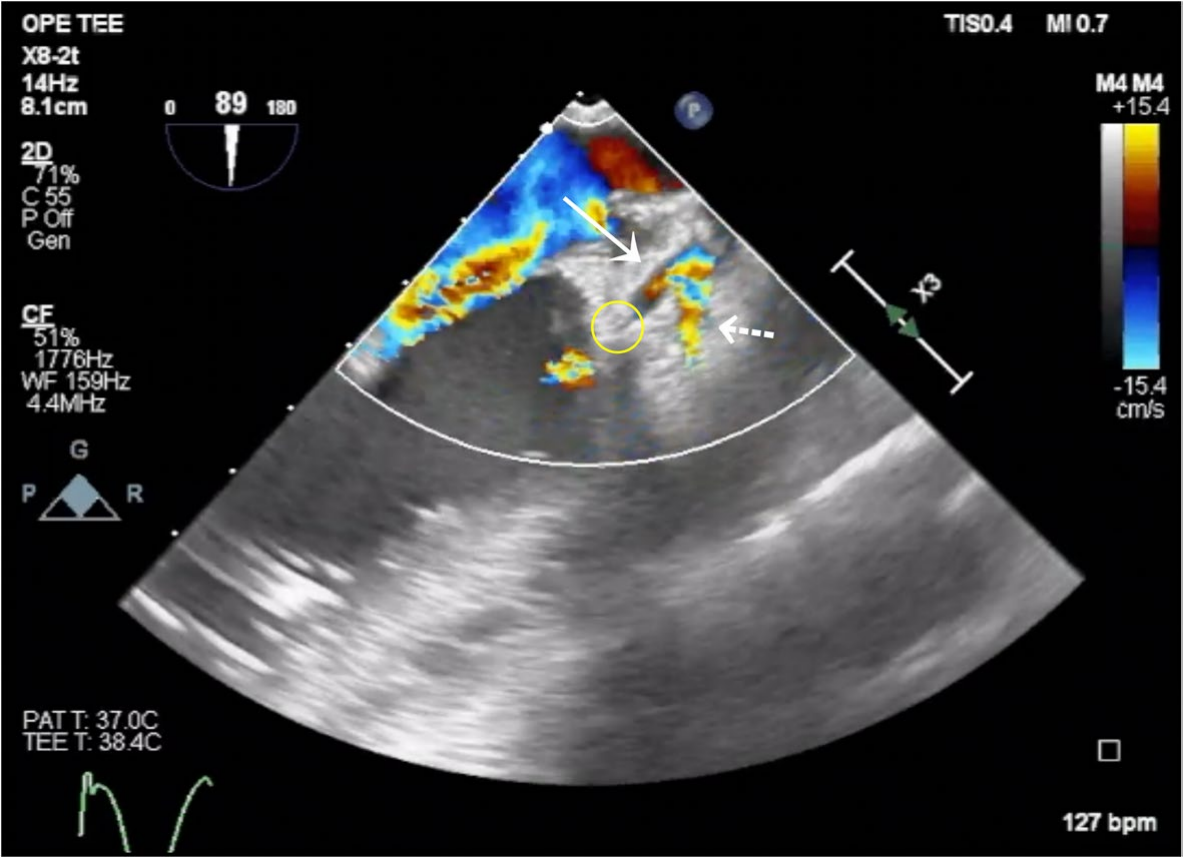
Flow interruption site in the left circumflex artery. Color Doppler image obtained using transesophageal echocardiography. The solid arrow indicates the left circumflex artery (LCx), and the dashed arrow indicates the left anterior descending artery (LAD). The yellow circle marks the site of flow interruption in the LCx

**Fig. 4:**
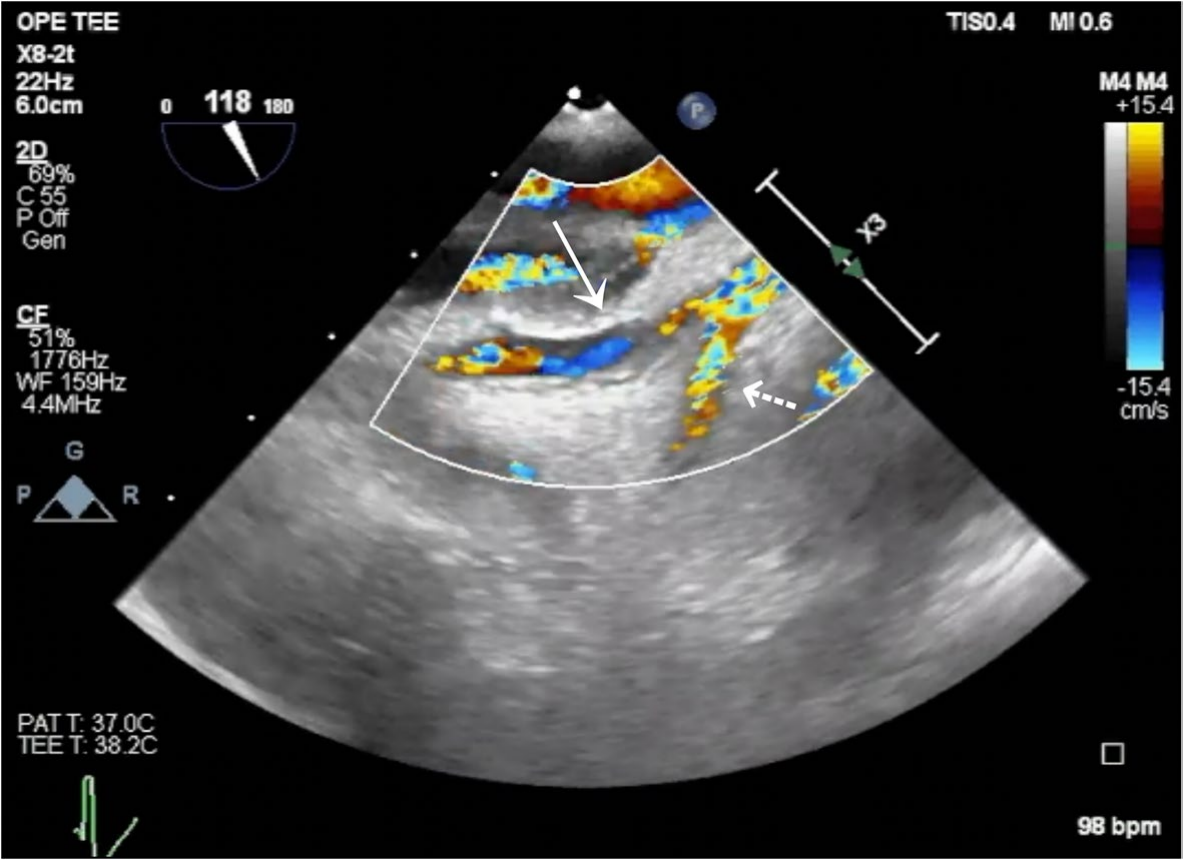
Restoration of flow in the left circumflex artery. Color Doppler image obtained using transesophageal echocardiography after removal of the implicated suture. The solid arrow indicates the left circumflex artery (LCx), and the dashed arrow indicates the left anterior descending artery (LAD)

Spontaneous cardiac activity resumed promptly following the administration of retrograde normothermic cardioplegia, indicating effective myocardial protection. ST-segment elevation in lead II was observed on electrocardiography. TEE in the mid-esophageal short-axis view revealed RWMAs involving the lateral and inferior myocardial walls (Fig. 1, Additional file 1: Video S1). Subsequent color Doppler interrogation demonstrated a complete absence of flow in the LCx (Figs. 2 and 3; Additional files 2 and 3: Videos S2 and S3). Based on the TEE findings, occlusion of the LCx secondary to the annuloplasty suture was strongly suspected and immediately relayed to the surgical team. Following removal of the implicated suture, restoration of coronary flow in the LCx was confirmed on TEE (Fig. 4, Additional file 4: Video S4). The patient subsequently developed ventricular fibrillation, which was terminated by defibrillation, restoring sinus rhythm. The ST-segment elevation on electrocardiography normalized, and the regional wall motion abnormalities resolved. Aortic regurgitation was effectively managed without the use of suture annuloplasty, and no residual stenosis was observed. The patient was smoothly weaned from CPB, and the procedure was completed without the need for blood transfusion. The total CPB time, total operative duration, and total anesthesia duration were 2 h 28 min, 4 h 56 min, and 6 h 26 min, respectively. The estimated duration of left circumflex artery occlusion was approximately 2 h. He was admitted to the intensive care unit under sedation and mechanical ventilation, extubated that evening, and transferred to the general ward on postoperative day 2. Follow-up transthoracic echocardiography on postoperative day 5 revealed no RWMSs and demonstrated preserved valve function without residual regurgitation or stenosis. A discharge plan was established based on clinical stability.

## Discussion

**Fig. 5:**
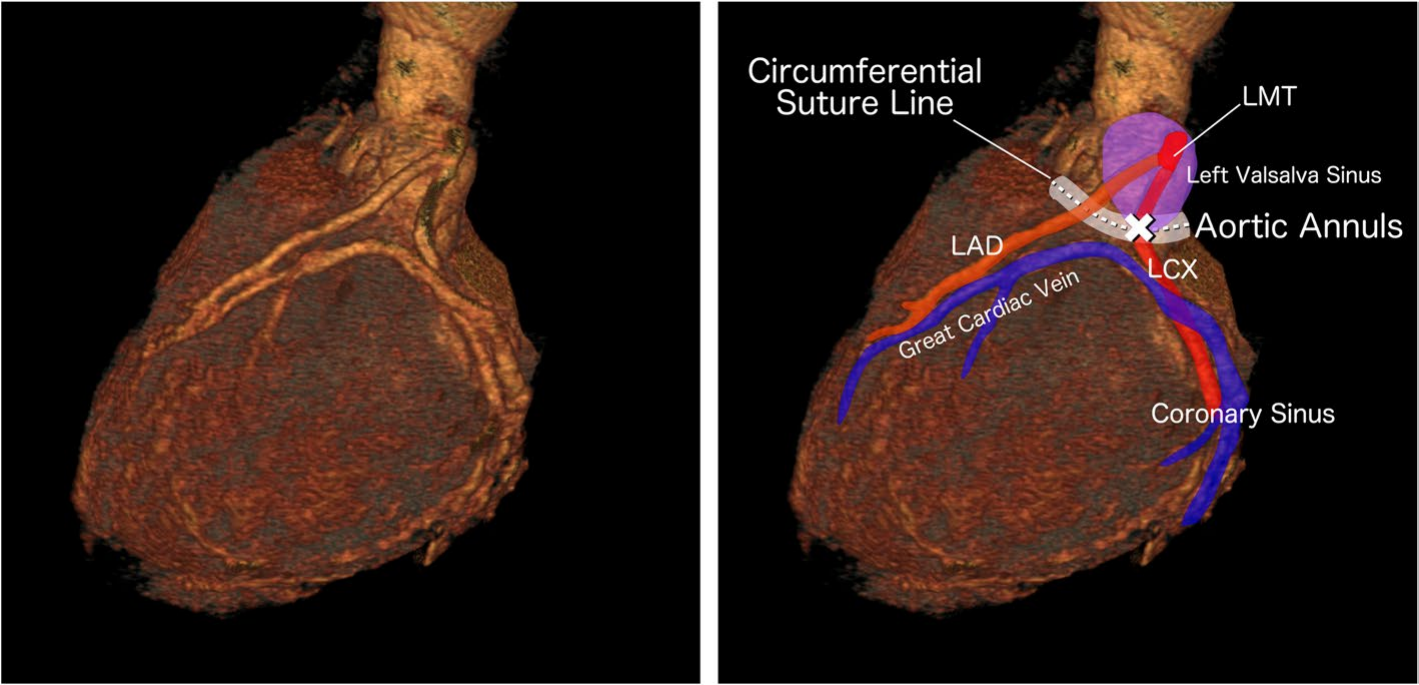
Three-dimensional computed tomography illustrating the presumed mechanism of left circumflex artery occlusion. The left panel shows the patient’s three-dimensional computed tomography image. The right panel provides a color-enhanced version, in which arteries and veins are highlighted in red and blue, respectively, and relevant anatomical structures are labeled. The white dotted line indicates the aortic annulus. The site of left circumflex artery occlusion is marked with a white cross

In the present case, intraoperative occlusion of the LCx occurred as a rare and unexpected complication of suture annuloplasty performed during aortic valvuloplasty. This technique entails the placement of a continuous circumferential suture along the aortic annulus to reduce its diameter and enhance leaflet coaptation [7]. To mitigate the risk of iatrogenic injury, neighboring anatomical structures—such as the LCx, right coronary artery, and the cardiac conduction system—are generally dissected bluntly and retracted with care to avoid entrapment in the suture line [7]. However, bicuspid aortic valve exhibits considerable morphological heterogeneity, characterized by variation in the number of raphes, commissural orientation, and leaflet symmetry [8, 9]. This marked anatomical diversity requires patient-specific approaches for surgical repair and generally contributes to higher technical complexity [8, 9]. Although no anatomical abnormalities were identified in the left coronary artery on preoperative imaging, the complexity of suture annuloplasty—combined with the anatomical challenges posed by the bicuspid valve—suggests that inadvertent LCx entrapment during annular suturing was the most likely cause of the occlusion (Fig. 5). Nonetheless, to our knowledge, no prior reports have described LCx occlusion as a direct consequence of this procedure, underscoring the exceptional nature of this complication.

Accurate intraoperative diagnosis of coronary artery occlusion using TEE hinges on meticulous preoperative evaluation and systematic archiving of high-resolution baseline images. At our institution, a comprehensive assessment of coronary flow is routinely performed and digitally preserved via TEE prior to the initiation of CPB. In the present case, direct comparison between real-time intraoperative findings and preoperative reference images enabled prompt and precise localization of the coronary obstruction, thereby facilitating timely surgical intervention. The diagnosis of LCx occlusion was substantiated by three converging lines of evidence: ST-segment elevation on intraoperative electrocardiography, RWMA identified on TEE, and complete absence of LCx flow on color Doppler imaging. While electrocardiographic alterations during open-heart surgery can be influenced by extrinsic factors such as lead positioning or cardiac manipulation, they remain a sensitive and readily accessible early marker of myocardial ischemia in the intraoperative setting. Detection of RWMA reportedly has high diagnostic accuracy for coronary artery disease [10, 11], and TEE is well established as a reliable imaging modality for this purpose. TEE, owing to its capacity for real-time visualization of cardiac morphology and intracardiac flow irrespective of intrathoracic orientation [12], enabled the intraoperative identification of LCx flow interruption through direct comparison with preoperative baselines—thereby substantiating the diagnosis of iatrogenic LCx occlusion.

Although intraoperative TEE enabled reliable real-time assessment of coronary perfusion, coronary visualization became limited during manipulation of the aortic root, as the root was exposed to ambient air. Supplementary intraoperative parameters might have allowed earlier recognition of evolving coronary compromise. During selective cardioplegia, perfusion pressures were continuously monitored in real time by the surgical team, although these values were not systematically recorded in the operative documentation. No significant pressure elevation was detected during subsequent antegrade cardioplegia; however, even subtle elevations in selective perfusion pressure, if present, might have provided an early indirect indication of coronary obstruction. In fact, retrospective review of intraoperative TEE images revealed that complete cardiac arrest was not maintained at certain time points, which may suggest that myocardial protection was suboptimal in this case.

In addition to diagnostic precision, intraoperative safety is greatly enhanced by the presence of mutual trust and effective communication among surgical team members. At our institution, this is actively cultivated through routine multidisciplinary communication, particularly between anesthesiologists and surgeons. In cardiac surgery, echocardiographic findings are critical for guiding anesthetic and hemodynamic management and for the early identification of surgical complications. In the present case, the anesthesiologist promptly detected absent LCx flow and relayed this finding to the surgeon, enabling timely recognition and resolution of suture-induced coronary occlusion. Prior studies have shown that strong collaboration among surgeons, anesthesiologists, and perfusionists can significantly reduce CPB duration [13]. Moreover, clearly defined roles and well-established interprofessional trust are essential for the early detection and prevention of complications [14, 15] and may positively influence perioperative outcomes [15].

Although LCx occlusion occurred during aortic valvuloplasty, meticulous preoperative and intraoperative assessment of coronary flow enabled prompt identification of the affected site and timely surgical intervention. The use of archived TEE images for coronary evaluation proved critical for confirming the diagnosis. Furthermore, strong interprofessional trust and effective communication among surgical team members played a pivotal role in ensuring procedural safety.

## Supplementary Information

Additional file 1: Video S1 Transesophageal echocardiography video in the mid-esophageal short-axis view showing a regional wall motion abnormality extending from the lateral to the inferior wall

Additional file 2: Video S2 Color Doppler transesophageal echocardiography video showing absence of flow in the left circumflex artery (LCx)

Additional file 3: Video S3 Color Doppler transesophageal echocardiography video showing the site of flow interruption in the left circumflex artery (LCx)

Additional file 4: Video S4 Color Doppler transesophageal echocardiographic video showing restoration of flow in the left circumflex artery (LCx) following removal of the implicated suture

## Data Availability

The datasets supporting the conclusions of this article are included within the article and its additional files.

## Abbreviations

CPB: Cardiopulmonary bypass
LAD: Left anterior descending artery
LCx: Left circumflex artery
RWMA: Regional wall motion abnormality
TEE: Transesophageal echocardiography
